# Glial cell mapping in the camel cerebellar cortex: a histochemical and immunohistochemical study

**DOI:** 10.1038/s41598-026-46231-4

**Published:** 2026-04-24

**Authors:** Abdelraheim H. Attaai, Ahmed E. Noreldin, Ahmed G. Nomir, Manal T. Hussein

**Affiliations:** 1 Department of Anatomy and Histology, School of Veterinary Medicine, Badr University in Assiut, Naser city, 11829 Egypt; 2 Department of Anatomy and Embryology, Faculty of Veterinary Medicine, Assiut University, Assiut, 71526 Egypt; 3 Department of Histology and Cytology, Faculty of Veterinary Medicine, Damanhour University, Damanhour, 22511 Egypt; 4 Department of Anatomy and Embryology, Faculty of Veterinary Medicine, Damanhour University, Damanhour, 22511 Egypt; 5 Department of Cell and Tissues, Faculty of Veterinary Medicine, Assiut University, Assiut, 71526 Egypt

**Keywords:** Camel, Cerebellum, Glia, Microglia, Astrocyte, Bergmann, Oligodendrocyte, Cell biology, Developmental biology, Neuroscience

## Abstract

The cerebellum is involved in numerous motor functions, the adaptation and execution of smooth complex voluntary and fine movements. Glial cells are essential for maintaining healthy neurons. Studying the camel cerebellum was neglected even in recent research even in the Middle East and Asia where camels are reared. Therefore, we investigated the glia in camel cerebellum employing histochemical and immunohistochemical staining using GFAP, Olig2, S-100 and Iba1 for astrocytes, oligodendrocytes, Bergmann glia and fibrous astrocytes, and microglia, respectively. The camel cerebellum was collected from ten clinically healthy mature camel’s (*Camelus dromedarius*) heads. GFAP immunoreactivity was not detected in the molecular layer, suggesting that this astrocytic population does not express GFAP. The distribution of astrocytes is heterogeneous between different layers and within the same layer. Bergmann glia was arranged into 4–6 rows in the Purkinje cell layer. Their processes extend radially through the molecular layer and reach the pial surface. Its mean density was 5126 cells/1 mm^2^. Oligodendrocytes were observed in the white matter and granular layer. The oligodendrocytes were either solitary or aligned in rows like beads along the nerve fascicles. Microglia were heterogeneous in both morphology and distribution in different layers and within the same layer. The white matter contains the highest mean density and they were elliptical and most processes run parallel to the nerve fibers. In the granular layer, elliptical- and oval bodies and their processes emerge from all over the cell bodies. The molecular layer contained oval microglia with short processes. Abundant Iba1 positive puncta distributed all over the molecular layer. The current study in camel is considered a step of comparative neuroglial biology across species.

## Introduction

The cerebellum, Latin for “little brain”, is a structure in the central nervous system. It is involved in various motor functions, including the adaptation and execution of smooth, complex voluntary movements (e.g., locomotion) and fine movements (e.g., reaching, grasping)^1^. It has been connected to higher-order functions, such as cognition, language processing, and emotional control^2^. For this purpose, the cerebellum communicates with several other neural regions, including the spinal cord^3^, the brainstem^4^ and the thalamocortical pathway^5^.

The peculiar laminar structure of the cerebellum is essential to its function^6^. An outer cerebellar cortex and an inner medullary core of white matter fibers with patched gray matter indicating the nuclei cerebelli of the camel comprised the camel’s cerebellum^7^. The white matter is covered by a three-layered cortex, consisting of an external molecular layer, a middle ganglionic (Purkinje cell) layer, and an inner granular^8^. Like most organs, brain physiology depends on “a village of cell types whose functions are relatively conserved across multiple tissues” ^9^. While neurons are important for neurotransmission in the CNS, glial cells are essential for maintaining healthy neurons. Numerous glial cell types are largely emphasized in the brain tissue, and each accomplishes specialized activities and has a distinct molecular signature^10^. These include supporting neurons’ metabolic needs, preserving rapid impulse conduction, regulating synapse development, maturation, and function, and controlling parenchymal homeostasis^11^. Depending on the species of mammals, glia cells comprise between 33 and 66% of the total brain mass^12^. Among these numerous cell types are oligodendrocytes, microglia and astrocytes, which are the close relatives of the Schwann cells, macrophages and stromal cells present outside the brain, respectively^9,13^. In addition, another type of neuroglia, Bergmann glia, is restricted to the cerebellum. This cell type is a specialized subtype of astrocyte (modified astrocyte), whose cell bodies occupy the Purkinje cell layer and sending their processes toward the pial surface^14^.

Microglia are considered the innate immune cells of the central nervous system and are also important participants in normal development and synaptic plasticity^15^. With growing interest in microglia during development, physiology and neurodegeneration, a better understanding of microglial biology as it has developed through evolution is important. Examining microglia from an evolutionary perspective is in its infancy and has been studied in a limited number of model organisms so far. Among mammals, our understanding of microglial cells largely originated from studies in rats and mice^16^. We have previously studied the neuronal elements in the camel cerebellar cortex^17^. To gain a better understanding of the distinct profiles of neuroglia across the central nervous system in various beings, more work is still required, even with the growing efforts of glial researchers. There are very few studies on the various glial cells in the adult camel’s cerebellum in literature. Hence, we aimed in the present study to investigate the histological, immunohistochemical and morphological characterization of the camel cerebellum glia to provide a detailed overview of the glial cells in the camel cerebellum. This may be able to elucidate the basis for regional vulnerabilities to CNS disease and disorders in the future.

## Results

The vermal region of folia VI-VIII of the camel cerebellum has the classical trilaminar structure constituting the mature cerebellar cortex. The intermediate layer is made up of Purkinje neurons somata and is positioned between an inner granule layer and an outer molecular layer. To obtain a general overview of glial cell intensities in the camel cerebellum, representative immunohistochemical staining for astrocytes, oligodendrocytes, Bergmann glia and microglia in adult camel cerebellum was applied.

### Astrocyte

#### Morphology

**Fig. 1:**
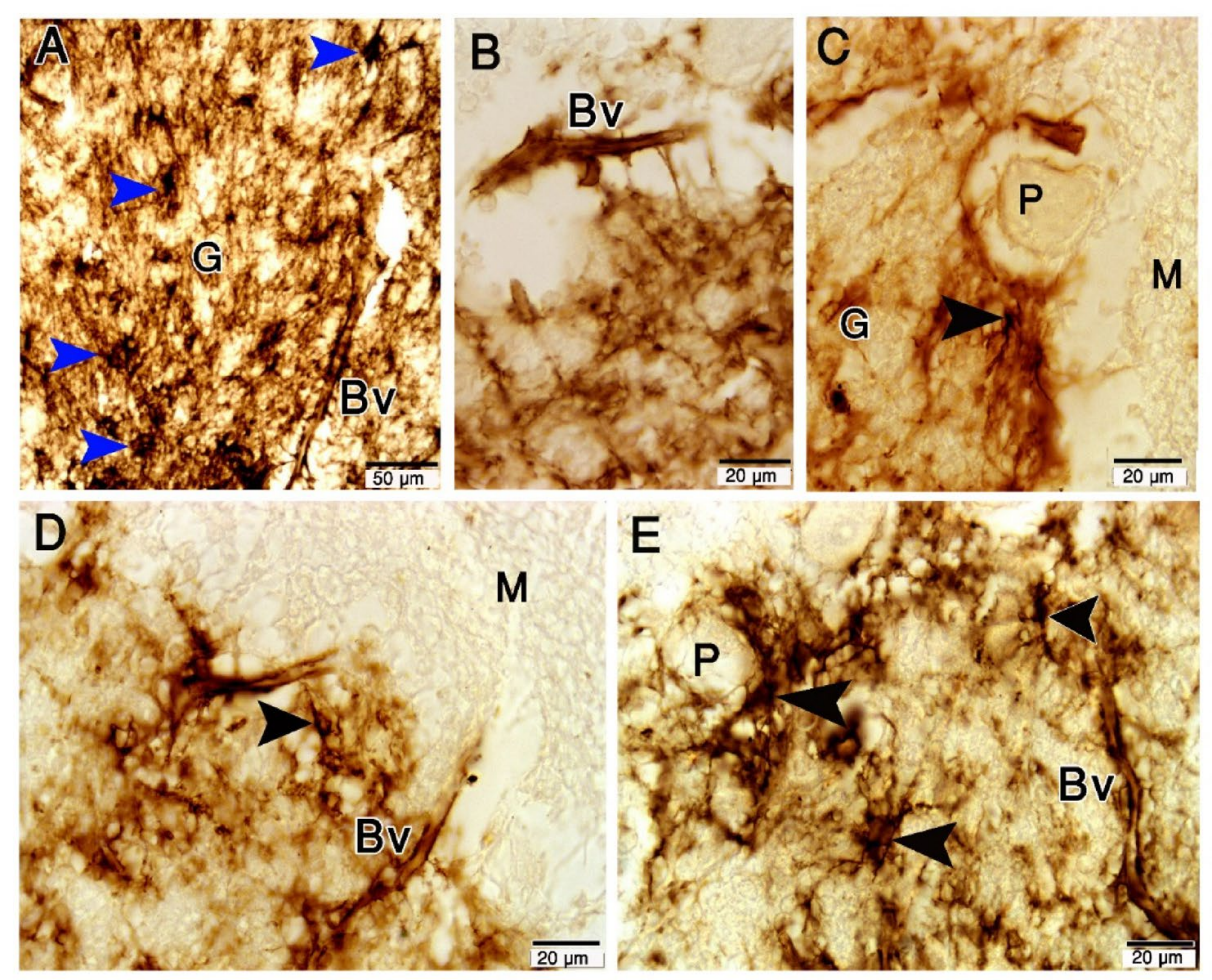
Paraffin sections show GFAP expression in camel cerebellum. (**A**): Astrocytes cell bodies (blue arrowheads) and their processes in the granular layer (G). (**B**–**E**): The Purkinje cell bodies (P) and the blood vessels (Bv) in the cerebellar cortex were associated with the astrocyte cell body and processes (arrowheads). The molecular layer (M) has very low or any GFAP expression. The scale bar A = 50 μm, B = 20 μm, C = 20 μm, D = 20 μm & E = 20 μm.

The Purkinje cell bodies and the blood vessels in the cerebellar cortex were intimately associated with the astrocyte cell body and processes (Fig. 1A-E). Camel astrocytes are morphologically classical and made up of a star-shaped soma and many processes, which are further divided into branches, branchlets, and leaflets in decreasing order (Fig. 1A). Several processes were seen ejecting from the astrocytes’ cell bodies and encircling the PCs and blood vessels (Fig. 1). Astroglia can be seen appended to the blood vessel wall in the cerebellar granular layers and medulla (Fig. 1).

Astrocytes are typically divided into two primary morphological groupings based on distinctions in anatomical locations and cellular morphologies: protoplasmic astrocytes throughout all gray matter and fibrous astrocytes throughout the white matter.

**Fig. 2:**
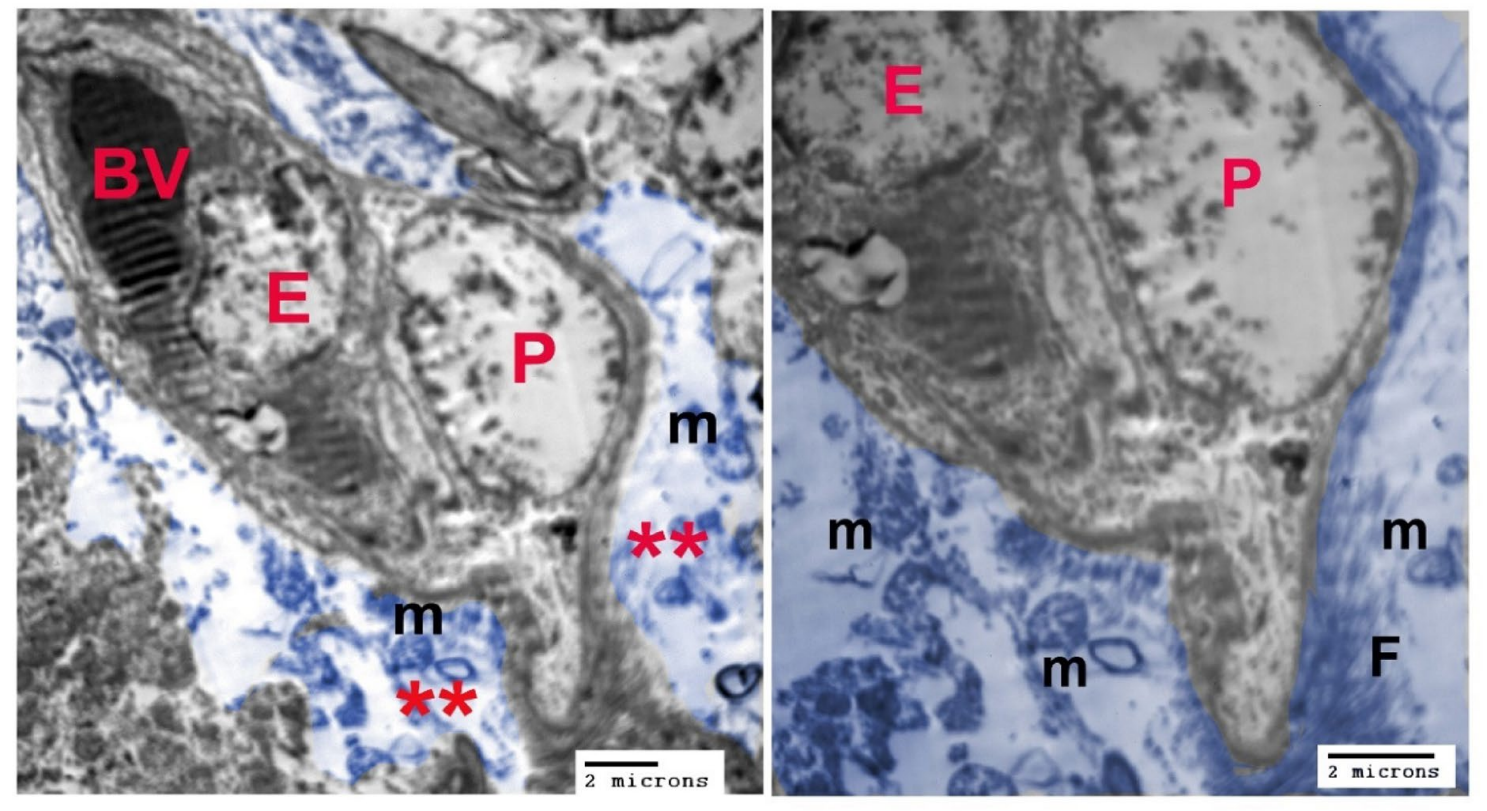
Colorized TEM images showing the blood brain barrier consisting of endothelial cell (E), pericyte (P) and astrocyte end-feet (blue colored regions, red asterisk) surrounding the blood vessels (BV), (M) mitochondria, (F) filaments.

The ultrastructure images show the blood-brain barrier, which consisted of the endothelium of the blood vessel, pericytes and astrocyte end-feet (Fig. 2A, B). astrocyte end-feet are often packed with mitochondria, which are often elongated and branched, lining the basal lamina of the microvasculature.

### Astrocyte distribution

**Fig. 3:**
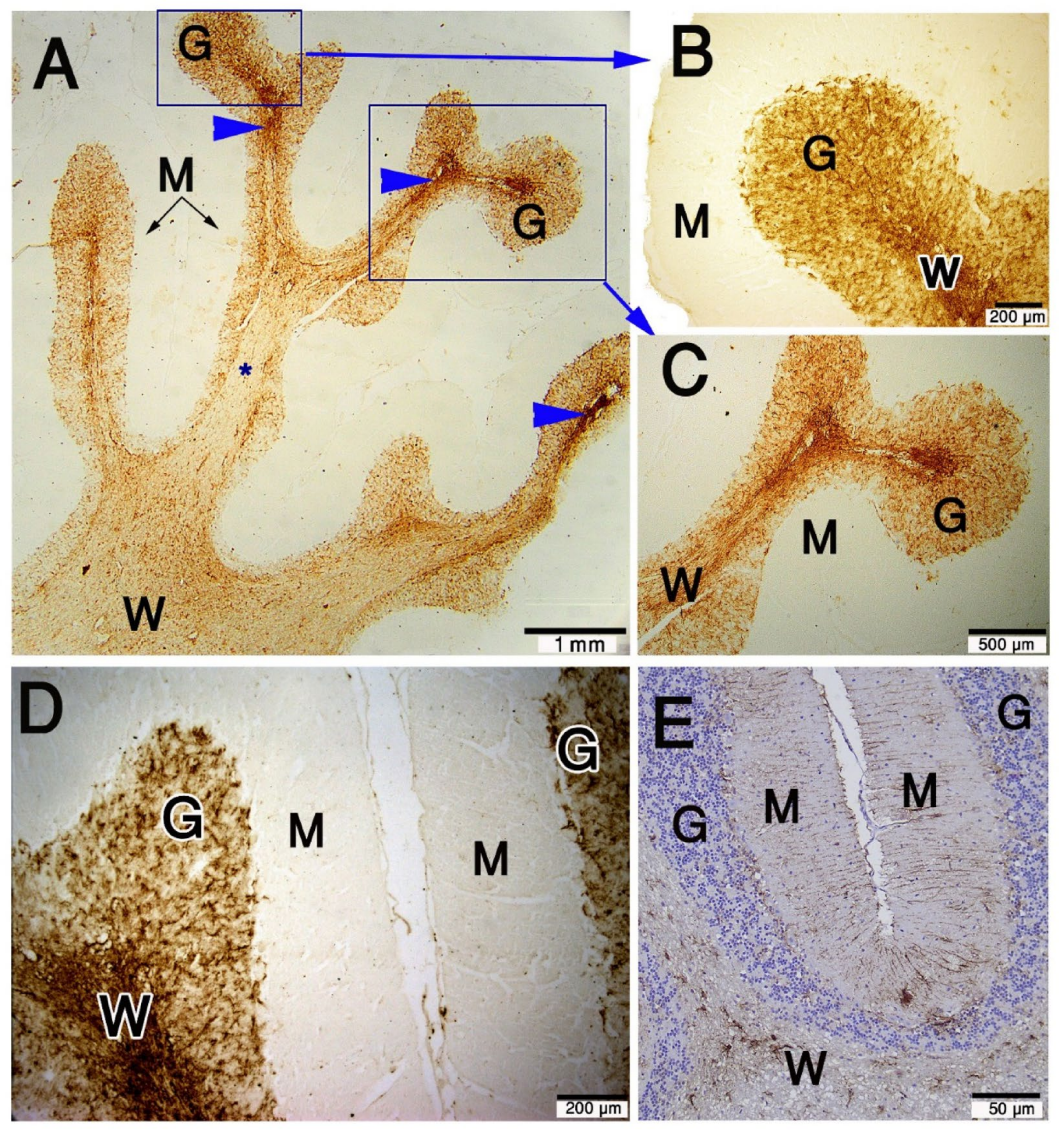
Paraffin sections stained with GFAP showing the expression in camel cerebellum (**A**–**D**) and rat cerebellum (**E**). (**A**–**C**): GFAP expressed in the astrocytes of the granular layer (G) and white matter (W). The molecular layer (M) is devoid of GFAP positive astrocytes or their processes. Some regions in the white matter have the heaviest density (blue arrowheads). (**E**): rat cerebellum, was used as a positive control section stained with GFAP antibody with camel sections, simultaneously, shows astrocytes in the molecular layer (M), granular layer (G) and white matter (W). The scale bar B = 200 μm, C = 500 μm & E = 50 μm.

In the current study, we demonstrated GFAP expression was localized in the cell body and thin processes of the astrocytes in the granular layer and Purkinje cell layer of the cerebellar cortex (Fig. 3A). The molecular layer lacked detectable GFAP-positive astrocytes, Bergmann glia nor their processes, which was obvious also when the hematoxylin counterstaining was not applied (Fig. 3A-D).

**Fig. 4:**
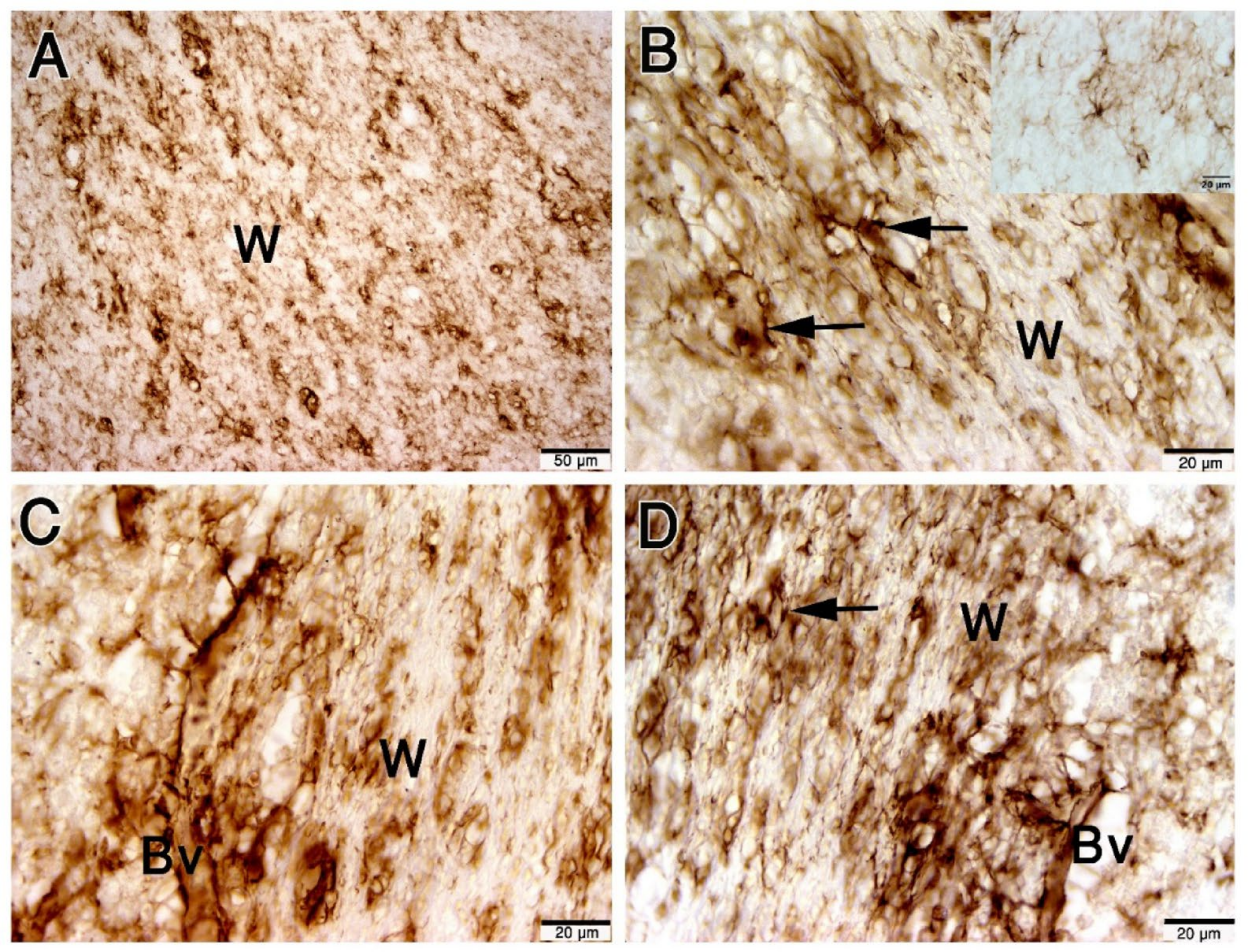
Paraffin sections immunostained with GFAP showing the astrocytes in cerebellum white matter (W). (**A**–**D**): GFAP expressed in the astrocyte cell bodies and processes (arrows) and appended to the blood vessel wall (BV). Scale bar A = 50 μm, B = 20 μm, C = 20 μm, D = 20 μm.

The distribution of astrocytes is heterogeneous between different layers and within the same layer (Table 1). There are some regions that have the heaviest density, especially at the summit of the cerebellar folia. The percentage of the area covered by the GFAP immunoreactive cells and their processes reached 78% in these regions. In addition, we noted that GFAP was expressed in the cerebellar medulla (white matter), which has the greatest heterogeneity, where the astrocytic coverage ranged from 1 to 78% in different regions. There are some areas at the proximal part of the cerebellar folia, which have nearly no astrocytes, where the coverage area reached 1%. Astrocyte density appeared higher near regions of aggregated granular cells, particularly at the summit of folia, and at the aggregated granular cells at the sides of folia. In the granular layer, the astrocyte density does not reach the highest density of the medulla. However, the heterogeneity in the granular layer is less than the medulla, where the astrocytic coverage ranges from 4 to 26% (Fig. 4A–D). One-way ANOVA showed a significant difference in GFAP coverage between layers (F(1,20) = 8.22, *p* = 0.0095). Tukey’s post hoc analysis confirmed significantly higher GFAP coverage in the white matter compared with the granular layer (*p* = 0.0095).

**Table 1:** The area coverage percentage of GFAP expression in the granular layer and white matter, using the ImageJ program.

	White mater	Granular
Minimum	1	4
Median	32	9.9
Maximum	79	26
Mean	35	12
Std. Deviation	24	7.9
Std. Error	6.9	2.5

### Bergmann cells

#### Morphology

**Fig. 5:**
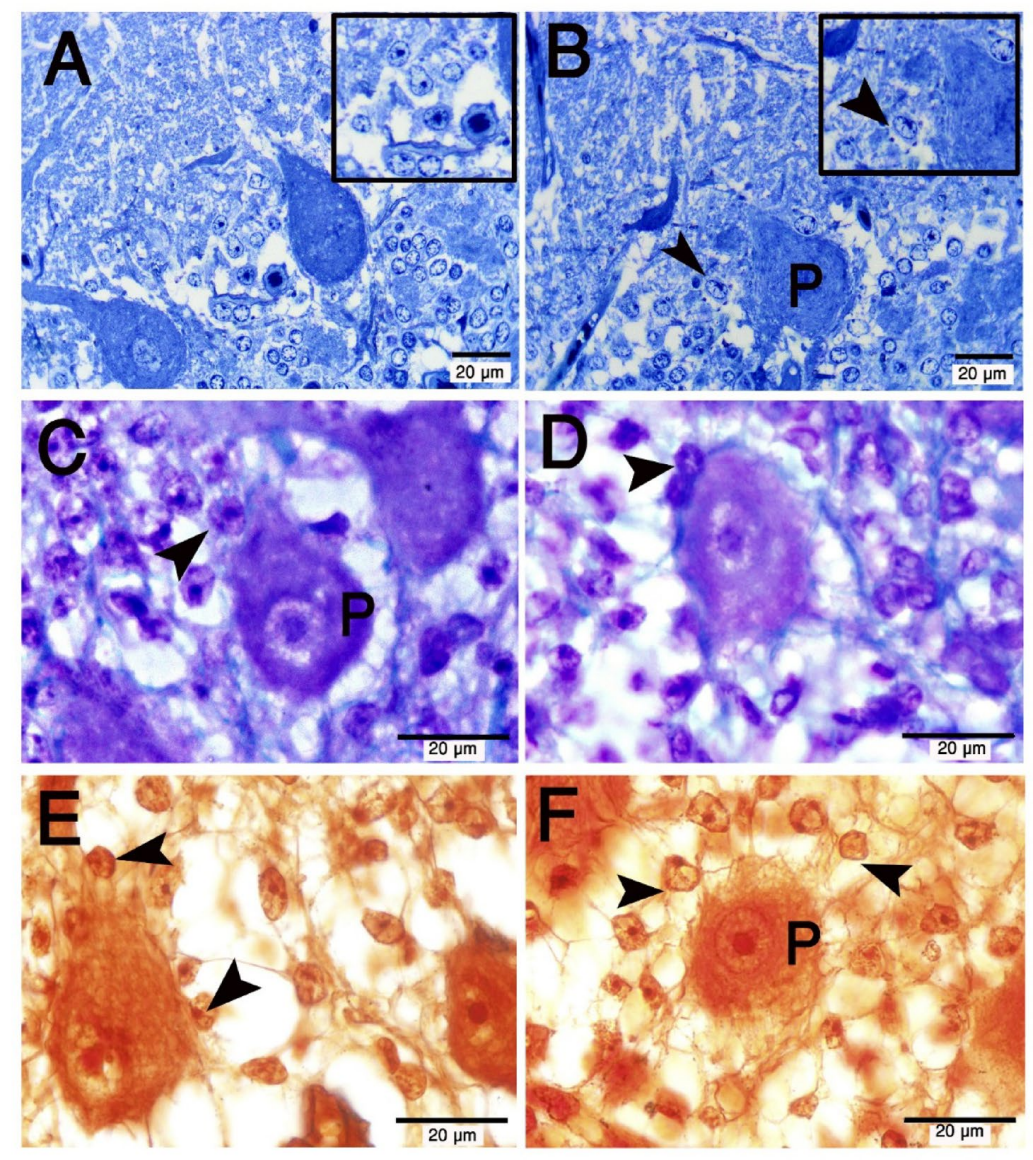
Morphology of Bergmann glia in the Purkinje cell layer (P) of the camel cerebellum. Semithin sections stained with toluidine blue (A, B), Paraffin sections stained with Cresyl violet and luxol fast blue (C, D) and silver impregnation (**E–F**) **A**. The cell bodies of Bergmann glia (arrowheads) are round or oval among the Purkinje cell bodies (P). There are delicate processes connecting Bergmann glia and Purkinje cells. Some Bergmann glia contacts the Purkinje cells directly. All scale bars are 20 μm.

Bergmann cells (BGCs) are unipolar cerebellar modified astroglia cells. We used 2 specific histochemical stains, (1) silver stain and (2) cresyl violet together with Luxol fast blue. In the Purkinje cell layer, we observed the BGCs arranged in rows, wrapping or even closely attaching to the Purkinje cell bodies (Fig. 5A-F). Delicate processes were extended from the BGCs cell bodies to contact BGCs and Purkinje cell bodies (Fig. 5C, E, F).

**Fig. 6:**
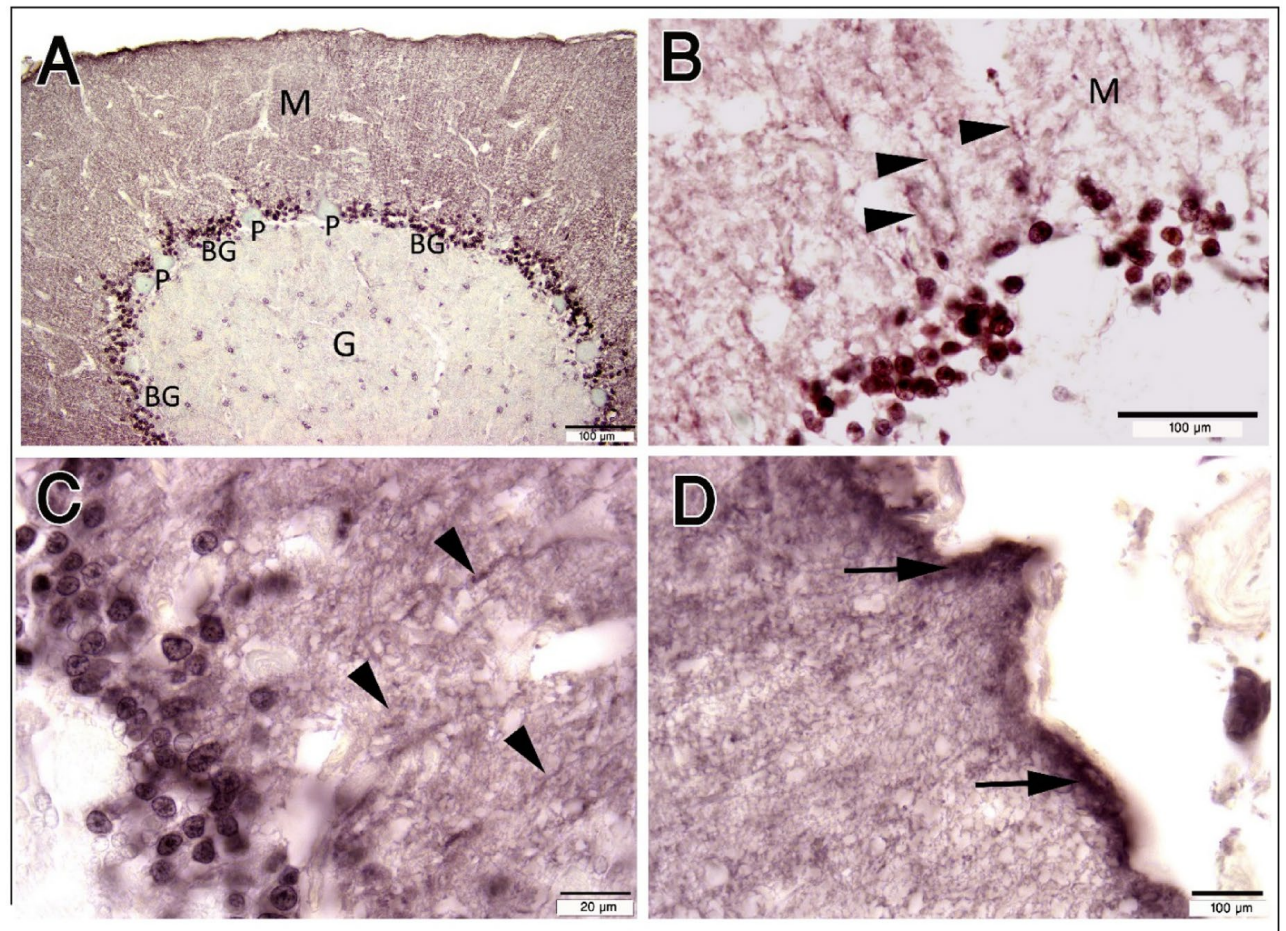
Paraffin sections immunostained with S-100 protein showing its expression in the Bergmann glia (BG) of the cerebellar cortex. (**A**–**D**): BG arranged into 4–6 rows of strongly immunopositive cells in the Purkinje cell layer (P). BGCs’ radial processes extend radially (arrowheads) through the molecular layer (M) until they reach the pial surface (arrows). G; granular layer. Scale bar A = 100 μm, B = 50 μm, C = 20 μm, D = 20 μm.

S-100B protein is frequently used as a marker for Bergmann glia and fibrous astrocytes in the cerebellum. BGCs’ radial processes extend radially through the molecular layer until they reach the pial surface (Fig. 6D). The length of the BGC processes equals the mean thickness of the camel molecular layer (323 ± 28 μm). The shaft fiber, which reaches the pial surface of the cerebellum, defines the molecular layer and is distributed throughout it (Fig. 6).

#### Distribution

BGCs are arranged into 4–6 rows of strongly immunoreacted cells against the S-100 protein in the Purkinje cell layer of the camel cerebellum (Fig. 6A–D). The crowded BGCs’ bodies reside in the Purkinje (ganglionic) cell layer, which contains a high mean density of BGCs, reaching 5126 cells/mm^2^.

**Fig. 7:**
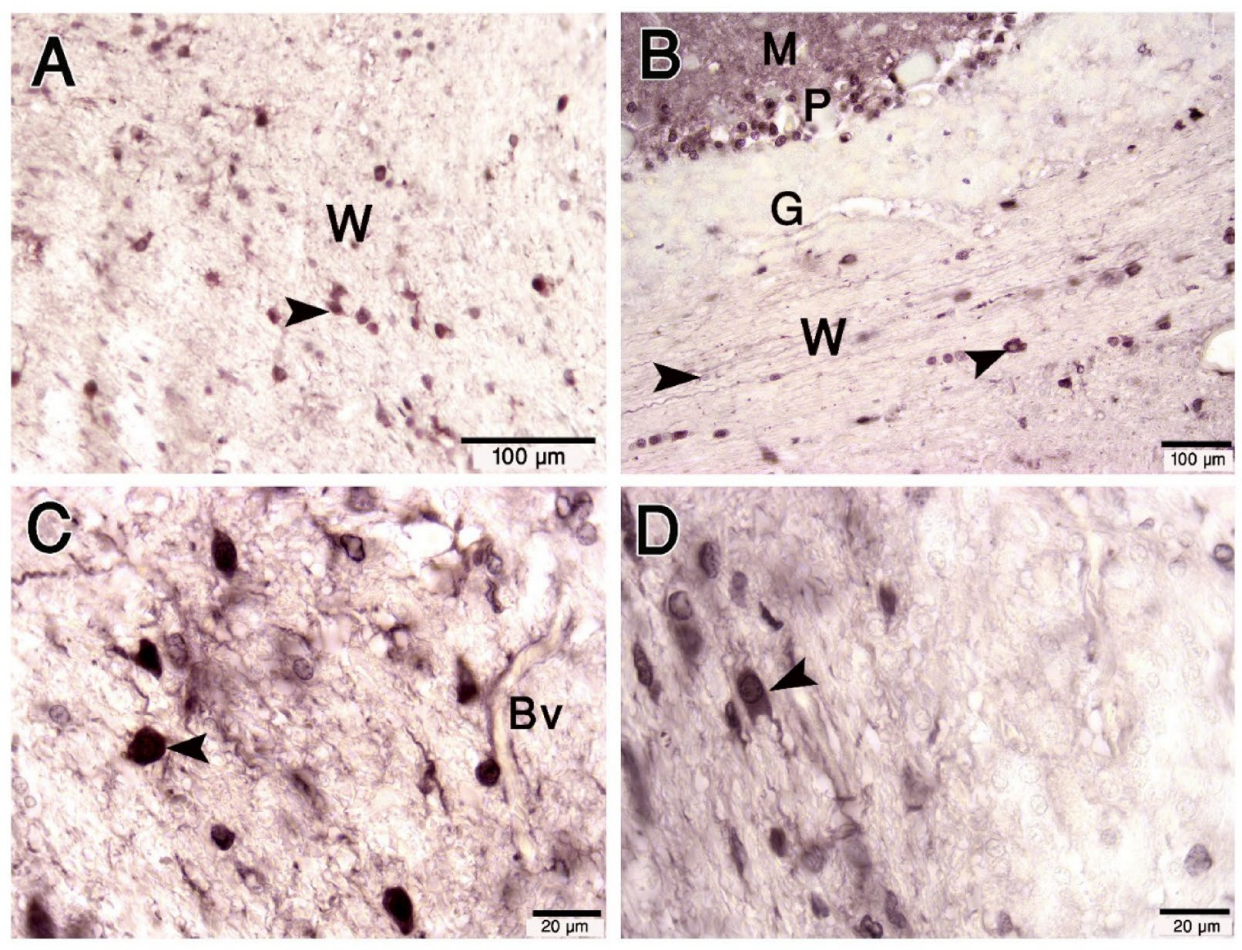
Paraffin sections immunostained with S- 100 protein showing the expression in cerebellum white matter (W). (**A**–**D**): Immunopositivity was observed in the cell body and processes (arrowheads) in the white matter (W) and were tightly associated with blood vessels (Bv). G: granular layer. Scale bar A = 100 μm, B = 50 μm, C = 20 μm, D = 20 μm.

Additionally, we showed that the fibrous astrocytes in the cerebellar white matter positively responded to the S-100 antibody. Their cell body and processes were observed in the white matter of the cerebellum. (Fig. 7A-D).

### Oligodendrocytes

#### Morphology

**Fig. 8:**
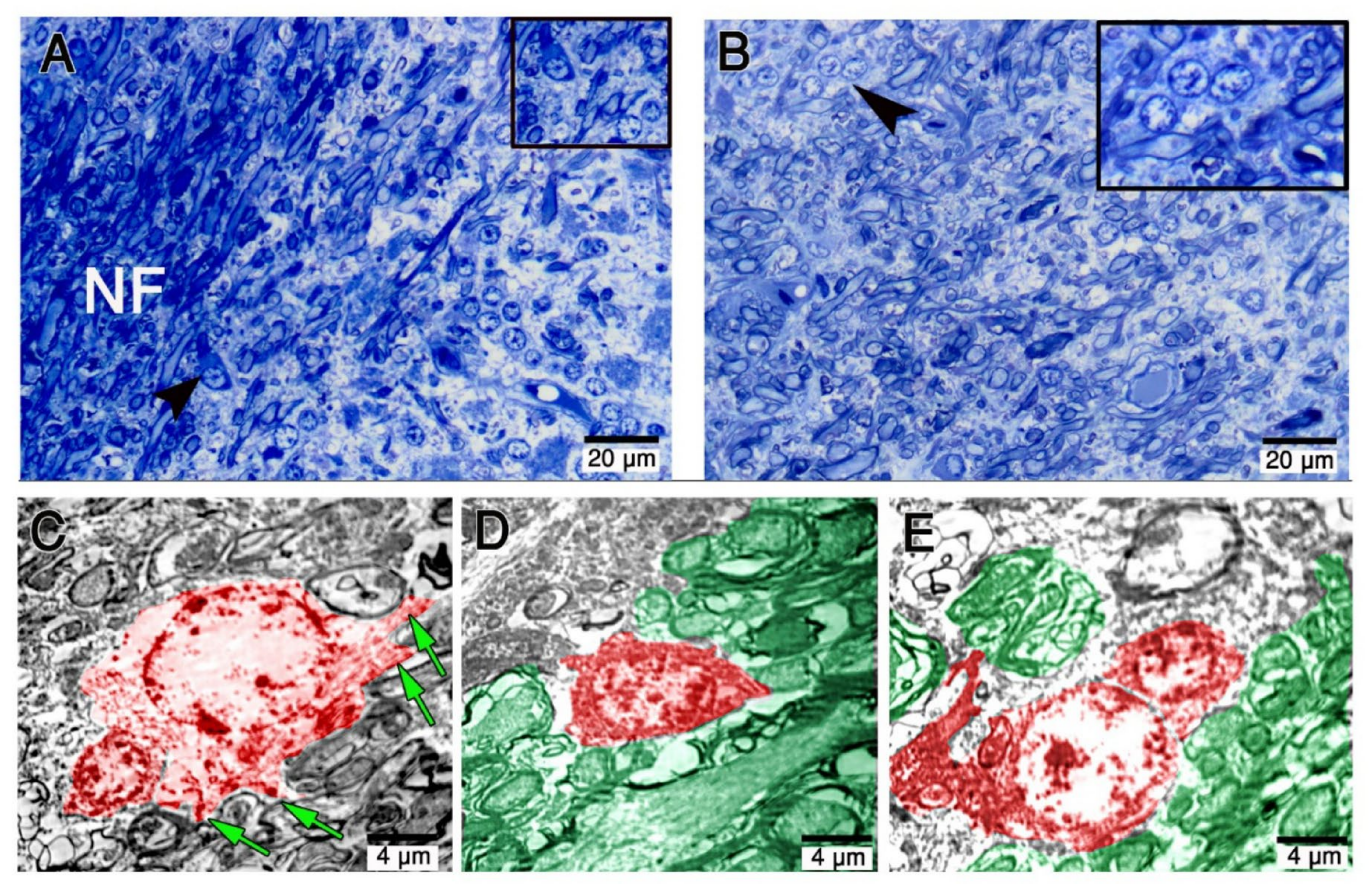
(**A**, **B**): Semi-thin sections stained with toluidine blue showing oligodendrocytes have large cell bodies with euchromatic nuclei (Insest). (**C**–**E**): Digitally colorized Transmission Electron microscopy TEM images showing oligodendrocytes (Pink) extending processes (green arrows) and the closely associated nerve fibers (green). Scale bar A = 20 μm, B = 20 μm, C = 2 μm, D = 2 μm & E = 2 μm.

The oligodendrocytes were visible, in the semi-thin section stained with toluidine blue, close to the myelinated nerve fibers in the white matter of the cerebellum (Fig. 8A, B). Ultrastructural analysis showed oligodendrocytes with large euchromatic nuclei, cell processes, mitochondria, and well-developed rough endoplasmic reticulum (Fig. 8C, D). Myelin sheath is a distinct structure that can be an ultrastructural hallmark of oligodendrocytes (Fig. 8C-E).

**Fig. 9:**
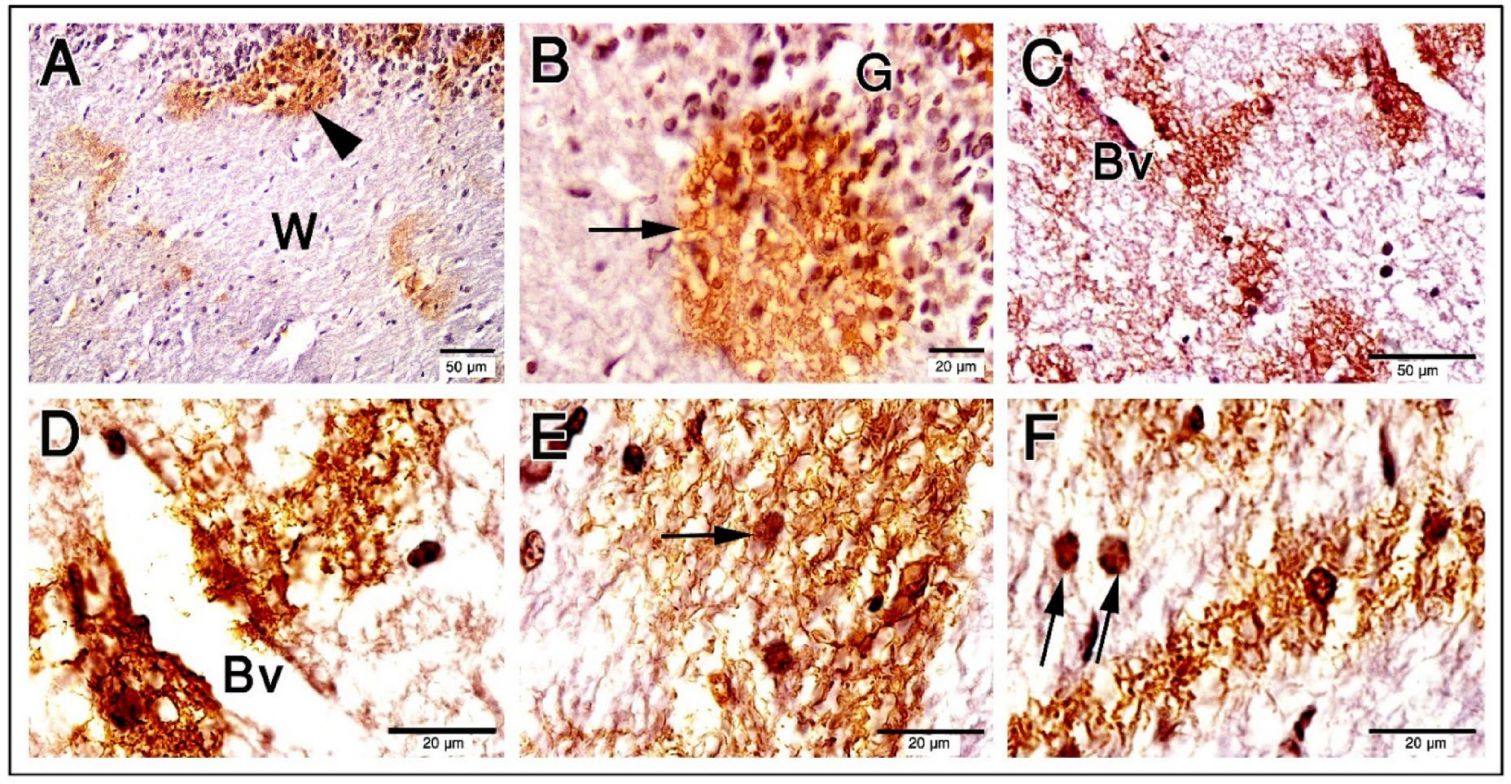
Paraffin sections immunostained with Olig2. (**A**–**F**): Olig2 expression was heterogeneous in the granular layer (G) and white matter (Me) of the camel cerebellum. The nuclei of oligodendrocytes are regular shaped round oval (arrows). Some Olig2 immunoreactive cells were abutting blood vessels (Bv). Scale bar A = 50 μm, B = 20 μm, C = 50 μm, D = 20 μm, E = 20 μm & F = 20 μm.

Olig2 expression was observed in the deep granular layer and white matter of the cerebellum (Fig. 9A, B). The nuclei of oligodendrocytes are regular-shaped round to oval, and relatively dark (Fig. 9C-F). Furthermore, Olig2 immunoreactive cells were abutting blood vessels (Fig. 9C, D). Olig2 expression was observed in the cell bodies and the processes that surround the nerve fibers of the camel cerebellum (Fig. 9E, F).

#### Distribution

**Fig. 10:**
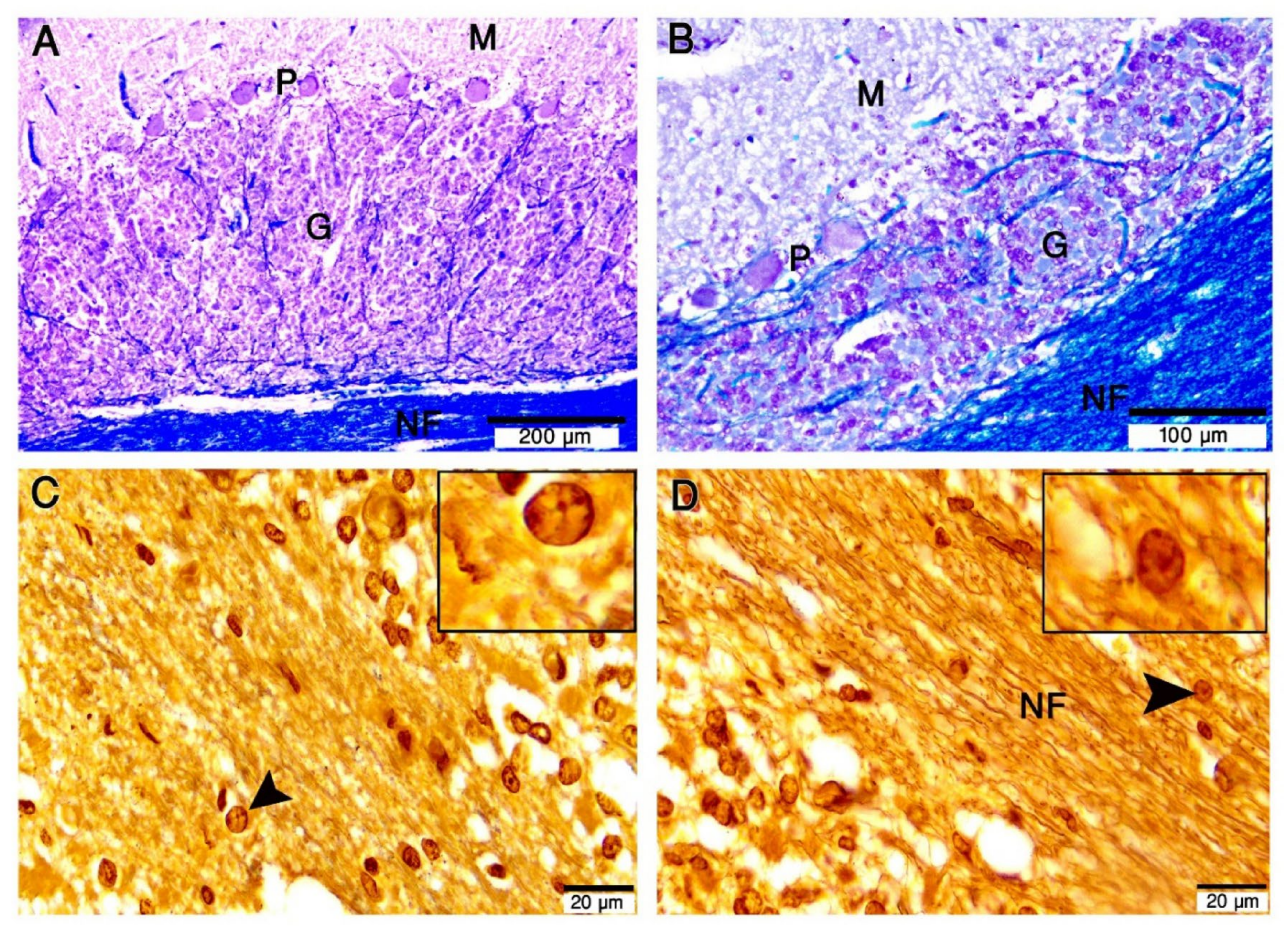
Paraffin sections stained with Luxol fast blue and cresyl violet (**A**, **B**) and silver impregnation (**C**, **D**) showing the camel cerebellum. (**A**, **B**): Many myelinated axons (blue) traverse through the granular layer (G) from the Purkinje cell layer (P). The myelinated nerve fibers (NF) in the white matter (blue color) of the cerebellum. (**C**, **D**): Silver stains showed the fibers in the white matter of the cerebellum and the large vesicular cell bodies of oligodendrocytes (arrowheads, insets). Scale bar A = 200 μm, B = 100 μm, C = 20 μm, D = 20 μm.

Luxol fast blue staining showed the blue-stained white matter, and many myelinated axons traverse through the granular layer from the Purkinje cell layer and the molecular layer, either individually or in bundles (Fig. 10A, B). The silver stain showed the fibers in the white matter of the cerebellum and the large vesicular cell bodies of oligodendrocytes (Fig. 10C, D).

### Microglia

#### Morphology

**Fig. 11:**
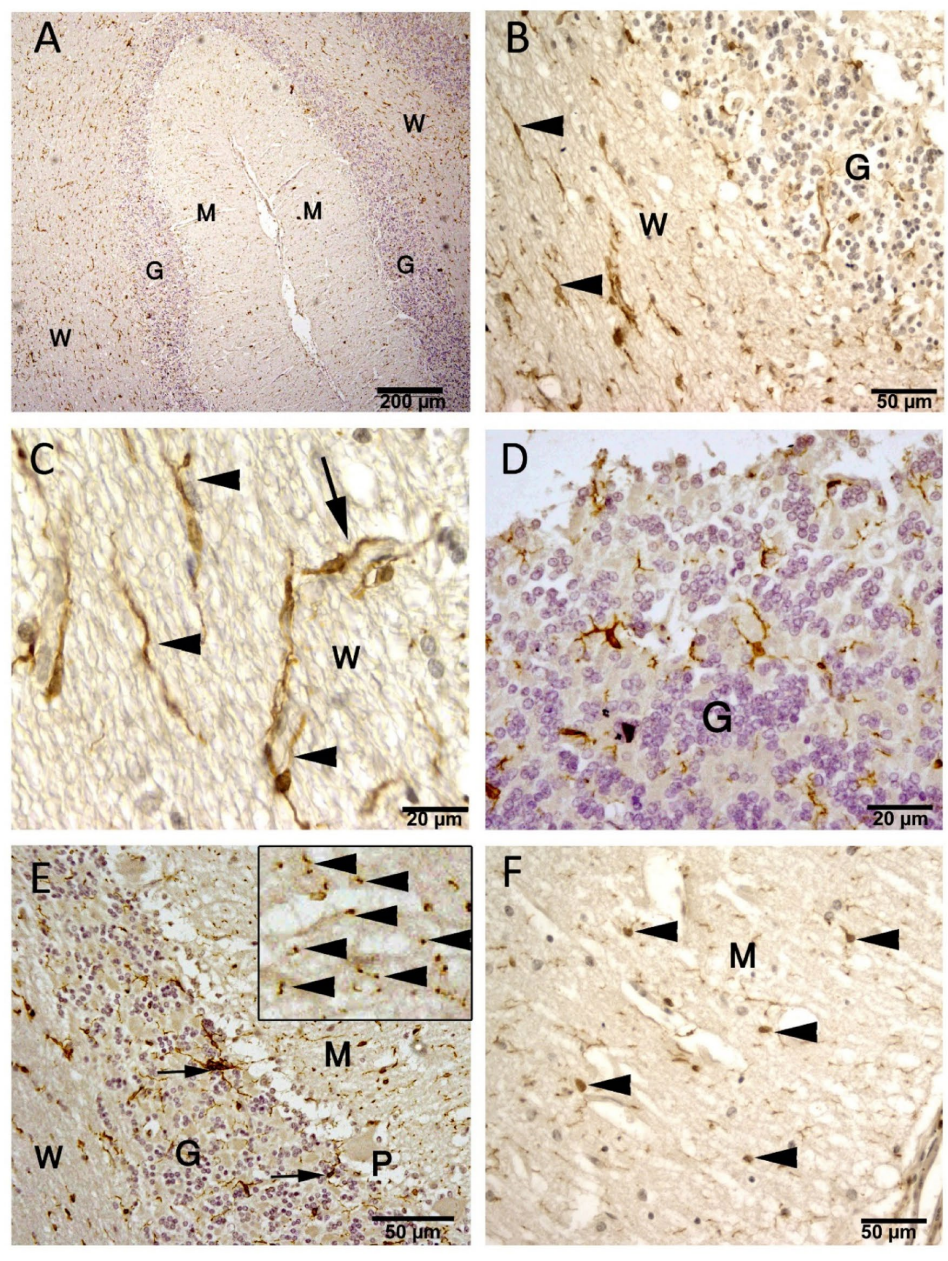
Paraffin sections immunostained with Iba1 showing microglia morphology in different cerebellar layers. (**A**) an entire view of the 3 cortical layers and medulla. (**B** & **C**) In the medulla, the microglia have an elliptical shape, and their processes run mostly parallel (arrowheads), or sometimes perpendicular (arrows), to the direction of the medullary nerve fibers. (**D**) In the granular layer, microglia have an oval shape, and their processes can be seen in any direction. (**E**) In the Purkinje cell layer, they are oval in shape, and their processes are directed toward the Purkinje cells. The inset shows many Iba1-puncta distributed all over the molecular layer. (**F**) In the molecular layer, microglia have a more rounded profile and fewer processes.

Microglia exhibited morphological and spatial heterogeneity across the cerebellar layers in different layers of the camel cerebellum (Fig. 11A). Morphologically, we noticed that in the medulla, the microglia have almost elliptical-shaped cell bodies. Their processes emerge mainly from the two poles of the cell bodies and run mostly parallel, or sometimes perpendicular, to the direction of the nerve fibers (Fig. 11B, C). In the granular layer, the microglia have both elliptical- and oval-shaped cell bodies. The processes emerge from all over the cell bodies and can be seen in any direction between the granule cell bodies (Fig. 11D). In the Purkinje cell layer, the microglia are mostly oval. Most processes are directed toward the Purkinje cell bodies, especially those emerging from microglia lying towards the granule cell layer (Fig. 11E).

In the molecular layer, oval microglia are the most frequent shape. The more superficial microglia have a more rounded profile. Most investigated cells in the molecular layer have one process that runs for a short distance and disappears quickly. However, many processes appear as puncta distributed all over the molecular layer (Fig. 11F).

#### Distribution

Camel cerebellar microglia were distributed heterogeneously in different layers and within the same layer. Microglia density in the cerebellum was 178 ± 57 cells per square millimeter (mm^2^). We found that microglia are distributed at different densities in different layers of the camel cerebellum (Fig. 11A). The cerebellar white matter contains the highest mean density in the cerebellum, with a mean of 205 ± 48 cells/mm^2^ and a median of 198 cells/mm^2^ (Table 2). In the cortex, the granular layer has the highest densities, with a mean of 179 ± 25 cells/mm^2^ and the molecular layer has the lowest density, with a mean of 88 ± 15 cells/mm^2^ (Table 2).

Second, there is a difference between the densities among different measured areas of the same layer. For instance, the minimal density in the granular layer was 140, whereas the highest recorded density was 201 cells/mm^2^. The white matter is the best example of the heterogeneous distribution, where the minimal and maximal densities were 128 and 279 cells/mm^2^, respectively. The molecular layer showed the lowest heterogeneous distribution, where the minimal and maximal densities were 73 and 103 cells/mm^2^, respectively.

Since the Purkinje cell layer appears linear in the sagittal section, the microglial densities were calculated in the case of the Purkinje cell layer as the linear densities per linear distance. The least densities among different cerebellar layers were demonstrated in linear densities with an average of 72 ± 44 cells, and a similar heterogenic distribution was also recorded with a minimal and maximal density of 28 and 115, respectively. The microglia found in the Purkinje cell layer were present mostly at the periphery of this layer, either beneath or above the Purkinje cell bodies.

**Table 2:** The mean densities of microglia in different cerebellar layers which were calculated per 1 square millimetre.

	White matter	Granular	Molecular	Purkinje
Minimum	128	140	73	28
Maximum	279	201	103	115
Median	198	183	88	74
Mean	205	179	88	72
Std. Deviation	48	25	15	44
Std. Error	15	11	8.7	25
P-value				

The counting was performed for at least 10 sections and the least number found was considered the minimum value and the highest number found was considered the maximum value which were the same results obtained from the Prism program.

**Fig. 12:**
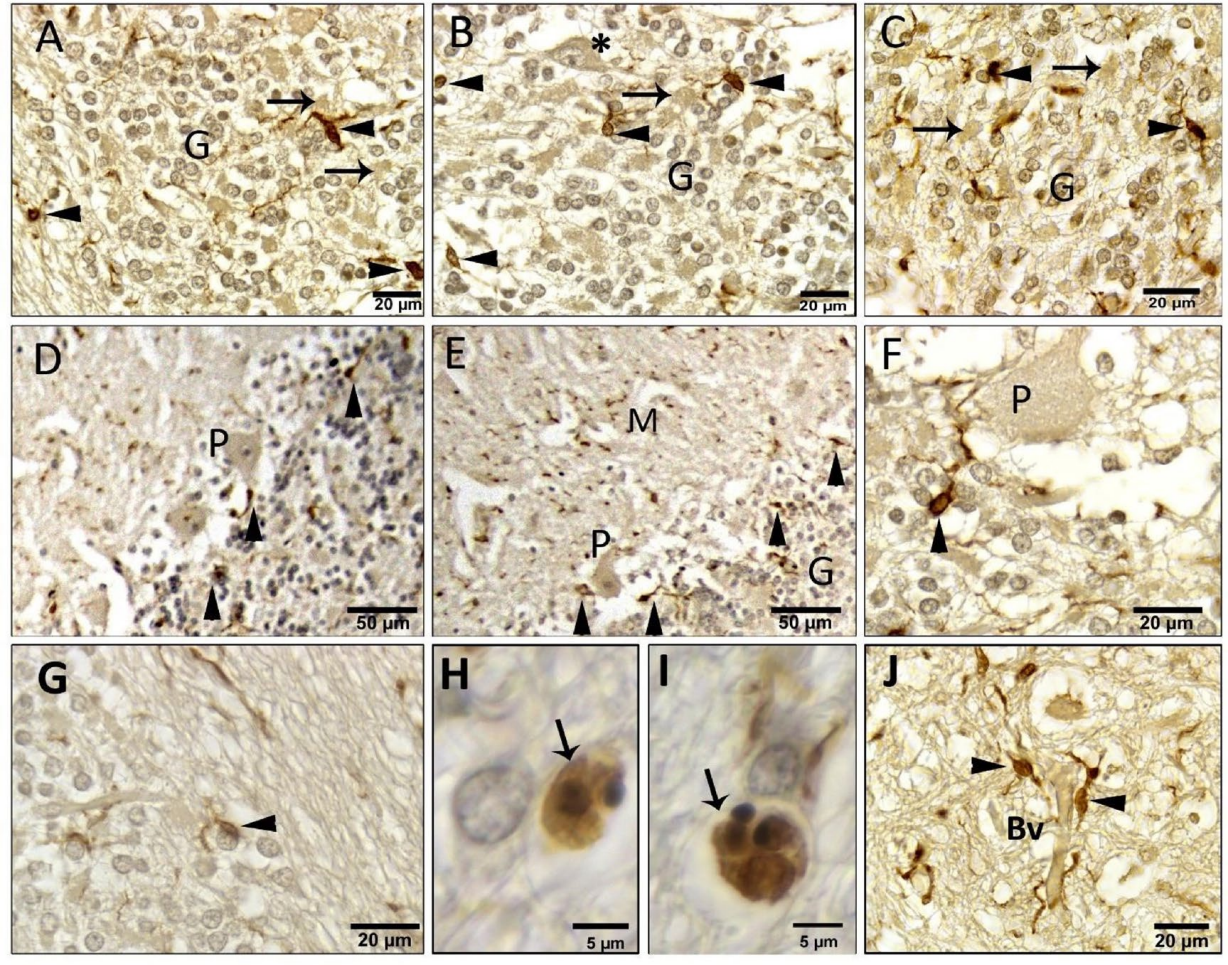
Paraffin sections immunostained with Iba1 showing (**A**–**C**). In the Granular layer, microglia (arrowheads) processes contact granular cells, Lugaro (asterisk) and glomerular rosette (arrows). (**D**–**E**) In the Purkinje cell layer, the microglia were either beneath or above the PC. (**F**) Microglia in the outermost zone of the granular layer were extending their processes to contact PC. (**D**–**E**) In the molecular layer, many Iba1 immunoreactive puncta appeared in the sagittal Sect.  13 (**G**) In the medulla, microglia and their processes were near the oligodendrocytes. (**H**, **I**) Some microglia were engulfing rounded apoptotic bodies. (**J**) The blood vessels were guarded by many microglia and their processes.

Since microglia continuously interact with their neighbors, we thought to characterize this interaction from the available pictures taken from different sections of the cerebellum. The cerebellar layers and cells could be identified easily by their unique shape and position. So, we could describe the contact between microglia and other cerebellar neurons, after staining microglia with Iba1. In the Granular layer, microglia were lodged between the granule cells. Microglial processes spread into different directions to contact surrounding granular cells, large non-traditional neurons and glomerular rosette (Fig. 12A-C). In the Purkinje cell layer, we noticed that the microglia of the PC layer in camel were mostly either beneath or above the PC. Microglia in the innermost parts of this layer were seen in direct contact with PC perikaryon (Fig. 12D-E). Sometimes, many microglia cells were seen encircling a single PC. Moreover, microglia in the outermost zone of the granular layer were extending their processes to reach and contact PC (Fig. 12F). Less contact was seen with the Bergmann glial cells. In the molecular layer, microglia were localized at low densities and were far away from the few molecular cells. We also observed many Iba1 immunoreactive puncta appeared in the sagittal section (Fig. 12D-E).

In the medulla, microglia and their processes were seen more frequently near the oligodendrocytes (Fig. 12G). Some microglia were engulfing rounded bodies just next to the oligodendrocytes (Fig. 12H, I). The blood vessels were guarded by many microglia and their processes (Fig. 12J).

One-way ANOVA revealed a significant effect of cortical layer on microglial density (F(3,17) = 12.44, *p* = 0.00015). Tukey’s post hoc test demonstrated significantly higher microglial density in the medulla compared with the molecular layer (*p* = 0.0019) and Purkinje layer (*p* = 0.0006). The granular layer also showed significantly higher density than the molecular (*p* = 0.030) and Purkinje layers (*p* = 0.010). No significant differences were observed between the medulla and granular layers (*p* = 0.646) or between the molecular and Purkinje layers (*p* = 0.963).

## Discussion

Understanding the form and structure of the CNS cellular elements is crucial for a better understanding of the pathogenesis of several CNS affections. Glial cells have been determined to constitute at least half of all cells and actively support brain function^11^. Astrocytes have been implicated in neuronal support and CNS homeostasis; however, their potential roles in camel cerebellar physiology require further investigation^18^. The variability of these cells also reflects the diversity of crucial functions that these glial cells perform for maintaining synapses, controlling synaptic plasticity, and contributing to the homeostasis of the brain^19,20^.

This study provides a detailed morphological characterization of glial cells in the camel cerebellum, which to our knowledge has been rarely addressed in previous literature. Regarding different glial cells immunostaining, we first thought that camel glia might not have the same molecules as those of the commonly used animals such as mice, rats, etc. and may not stain for the available antibodies. We found immunopositivity staining of camel glia cells to the used antibodies. These findings suggest that these molecular markers are likely conserved among diverse mammalian species^22^.

### Astrocytes

Camel astrocytes are morphologically similar to other mammals’ astrocytes^23,24^. In the camel cerebellum, the distribution of astrocytes varies between different layers, which likely contributes to their functional heterogeneity^25–27^. In the camel molecular layer, no GFAP+ astrocyte was encountered, although it has been reported in macaques and human (33%), mice (29%)^28^ and donkey (6%). Since the donkey cerebellum had weak GFAP immunoreactivity^26^, it might be the nearest one to the camel’s one among the available literature. The absence of GFAP immunoreactivity in the molecular layer likely reflects very low or any GFAP expression in astrocytic subtypes residing in the molecular layer in this species. Therefore, GFAP preferentially labels fibrous astrocytes and may not reliably detect all astrocytic subtypes, including Bergmann glia.

In the camel granular layer, the highest region of GFAP+ astrocyte area coverage (26%) were lower than that described in humans and macaques (34%) and mice (29%)^28^. Indeed, the mean GFAP+ astrocyte area coverage (12%) in camel is much lower than aforementioned organisms. The donkey cerebellum showed the highest degree of GFAP expression (57.20%) in the granular layer^26^. In white matter, although the mean GFAP+ astrocyte % area coverage in camel (35%) lies within the range described in mice 31%, in macaques 38%, and in humans 43%^28^, we observed robust regional differences in camel, where it ranged from 1 to 79%, which related proportionally to the number of granular cells next to them.

Immunohistochemical staining of the camel astrocytes showed their distal processes contact the blood vessels to generate perivascular end-feet^29^. The blood-brain barrier (BBB) is a multicellular structure made up of astrocytes, pericytes and endothelial cells^30^. BBB is characterized by a low vascular permeability that prevents the majority of blood-borne chemicals from entering the brain parenchyma^31^. The BBB’s integrity, as well as the preservation of ionic and water homeostasis, depends on the close connection between astrocytic end-feet and the vasculature^32^. Astrocytes have the capacity to up-regulate a variety of BBB characteristics, because of strong tight junctions, the expression and polarized localization of transporters^33,34^.

In fact, astrocytes perform a diverse range of tasks to support neurons both structurally and functionally^12^, including the uptake of neurotransmitters, recycling and trophic factor release, assistance in wound healing and regeneration, and synaptic density modulation^35^. Additionally, astrocytes have a high degree of plasticity and can change their morphology and function over the span of a lifetime^36^. Recent studies have shown that astrocyte dysfunction contributes to the development of neurological illnesses, including neurodegenerative diseases and their role in the ageing of the brain^37,38^.

### Bergmann cells

Bergmann glial cells in the camel cerebellum showed immunoreactivity to S-100 protein, which is a more sensitive marker for Bergmann glia in the cerebellum. However, unlike those in mice or rats, Bergmann glia did not respond in the same manner to GFAP^21^ and monkeys as well^39^. They are also not immunopositively to GFAP in birds^40^.

The camel Bergmann glia number (5126 cells per 1 mm^2^) outnumber the Purkinje neurons, however, their number still beyond the mean areal packing density of 8,269 somata/mm^2^ in rats^41^.

We found that the processes of Bergmann glia radially span the entire length of the molecular layer, which is classical for most classes of vertebrates higher than fishes. The mean thickness of the camel molecular layer was 323 ± 28 μm. This is nearly the same as that of man, which is thicker than other species^42^. The molecular layer measured 245 μm in rhesus monkey (*Macacca mulatta*), 160 μm in rat (*Rattus norvegicus*) and 70 μm in African mice (*Leggada minutoides*). The species with thick molecular layers (man, monkey) have thicker stem processes^42^. It has been suggested that larger animals with longer gestation periods allow for prolonged growth of cell volumes^42^.

The architecture of Bergmann glia with their processes spanning the molecular layer resembles the radial glia, from which it is believed that neural stem cells (NSCs) of the mature brain develop^43^. Bergmann glia also shares certain markers with radial glia and neural stem cells, like the L-glutamate/L-aspartate transporter (GLAST)^44^, the brain-specific lipid binding protein (BLBP)^45^, the calcium-binding protein S-100^46^, Sox1 and Sox2^47^, suggesting that these cells might be adult neural stem cells. During development, these radial processes act as guidance for granule neurons that migrate, and the terminal end-feet of these BG tile together to create the glia limitans that cover the cerebellum. Bergmann glia has also been suggested to play a role in the development of Purkinje cell dendrites and the stability of synaptic connections onto these neurons^42^.

Bergmann glial processes surround the synapses of Purkinje cells and contribute to proper synaptic transmission and synapse maintenance^48,49^. This involves the buffering of ions, the uptake of neurotransmitters and the creation of glutamine, which neurons then convert to glutamate^50^. The Bergmann glia cells interact with a wide variety of neuronal subunits and are involved in a number of processes that support the cerebellar circuits^14^. The Purkinje cell dendrites, synapses, and soma are intricately ensheathed by the Bergmann glial cells, which exhibit an intricate connection with Purkinje cells^51,52^. It has been demonstrated that the experimentally induced deletion of Bergmann glial cells in transgenic mice led to substantial impairments in cerebellar neurons and motor dyscoordination as a result of missing the glial-neuronal connections^53^.

### Oligodendrocytes

In the white matter of the camel cerebellum, the Olig2 immunoreactive oligodendrocytes were abundant and they are often arranged like beads on a string aligned along the fiber tracts, as has long been described before in rat cerebellum^54^. A century later, a similar description was reported by Pio Del Rio Hortega and Wilder Penfield in humans/mice using silver carbonate staining. They noted that oligodendrocytes were enriched in the white matter and also found in the grey matter, and were mostly localized interfascicular, sometimes also perineuronal or perivascular. The electron microscopic analysis revealed similarities between oligodendrocytes of camel and rats^55^.

### Microglia

The observed microglial cells in the camel cerebellum were visualized after staining with Iba1. In camel, as in all examined species so far, microglia consist of a central cell body emitting a number of branched processes^8,56–58^, with a varying degree of complexity across species, however, independent of complexity in phylogenetic evolution^56^. The differences in shape could be attributed either to the different neuronal architecture in different layers or the consequent different required functions^64^. The most common shape is the less branched bipolar shape with two main processes.

Although the common ramified microglial structure exists in most brain territories, they differ to some extent within the same anatomical region^60^. For instance, microglia in the cerebellum contained relatively smaller soma and bigger cytoplasm area, but lower ramification complexity and covered area than those in other anatomical entities^61^.

Camels’ microglia morphology was comparable to those of rats^59,62^. The morphological variations within a particular territory might be secondary to their location, i.e. microglial processes extend to fill the interstices in an array^62^. For example, the differences between the cortical and medullary microglia morphology could be attributed to the numerous biologically active substances in the grey matter which stimulate the growth of microglial processes, to increase the area of microglial interaction with the neuronal somata^59^.

In the current study, the mean density of Iba1-positive cells exceeds approximately three times, and six times the number of microglial cells in adult rat cerebellum detected using the lipocortin 1 antibody^59^ and in the murine brain using the F4/80 marker^62^, respectively. This huge difference could be attributed to either the different used markers, i.e. the more specificity of recent antibodies, or a real difference in camels than rats. It has been reported that the minimum density of microglia in the murine cerebellum, which could be related to its nature of receiving the majority of primary afferents^59^. Therefore, this could indicate a possible difference in the primary afferent to the camel cerebellum, which needs further investigation. Therefore, differences in microglial density may reflect methodological as much as biological variation.

The microglial density in the camel cerebellum varies among layers, with no significant differences between the different cerebellar lobules^63^. In the camel cerebellum, Iba1 immunoreactive cells in GL were higher than the ML/PCL, similar to the previous studies in both mice and rat cerebella^15,62,63^.

The camel cerebellar white matter contains the highest mean density, comparable to that noted in mice^62^. The molecular layer of the camel cerebellum was notable as the less densely populated microglia, like the mouse cerebellum^61,62^. These authors suggested that microglial arrangement may be regulated by a sort of spatial signal, such as the presence of some ligands, or inhibitory or modifying factors^62^. The large number of Iba1 immunoreactive puncta in the molecular layer might indicate that most processes emerge from a right angle to the Purkinje and Bergmann Glia processes. i.e. at the Z direction.

The distribution of Iba1-immunopositive cells within the same histological layer of the camel cerebellum was heterogeneous within the same layer of cerebellum, as previously detected in mice by either F4/80- or NDPase-positivity^61–63^. This was also reported where microglia are not tiling the Cb as completely as they do the cortex in the mouse cerebellum^15^. These researchers observed that the cerebellar microglia somata were motile with frequent rapid displacements, as recorded by the aid of a two-photon microscope. They suggested that their motility compensates for their lower number, lower ramified morphology and more complex cerebellar issues than the cortical one. This view is supported by their phenotypic characteristics, such as the higher expression of adhesion and cell migration genes^64,65^.

The microglia of the PC layer in camel were mostly either beneath or above PC. This has also been noticed in mice cerebellum^63^, however, these authors expressed, in other words, that there were no microglia in the PC layer. The presence of microglia in PCL has been proved in a more recent study, with more advanced techniques^15^.

The glia in camel cerebellar cortex and white matter showed some differences in their distribution and densities than humans and other animals. However, cross-species comparisons of glial densities are inherently limited by differences in: (1) tissue fixation and processing protocols; (2) antibody clones, species reactivity, and dilution factors; (3) section thickness and stereological vs. non-stereological counting methods; (4) the specific cerebellar regions sampled; and (5) biological variables such as sex, age, and body size. Comparative analysis of neuronal and glial elements across species could help elucidate evolutionary adaptations in cerebellar organization. More specific physio-morphological research is required to seek in depth the camel cerebellum to discover the cause of these differences.

One of the limitations was using mixed sex of animals randomly. Given that sex-related differences in glial density and morphology have been reported in rodent models, future studies should include sex as a biological variable to assess its potential influence on camel cerebellar glial organization.”

In summary, the camel cerebellum displays a conserved trilaminar organization with species-specific glial adaptations. These findings enrich the comparative neuroanatomical understanding of glial diversity among mammals. This study was based on a limited number of adult camel specimens obtained post-mortem from a local abattoir. Functional or molecular assays were not performed, and some antibodies were validated based on cross-reactivity rather than camel-specific epitopes. Future work incorporating transcriptomic and electrophysiological analyses is needed to clarify the functional significance of these observations.

## Materials and methods

### Collection of specimens

The total number of camels (*n* = 10) were slaughtered humanely in accordance with Egyptian abattoir regulations and the local ethical guidelines for animal handling. All camels were healthy adult animals that were slaughtered for meat production, and the brain samples were collected immediately post-mortem. No animal was euthanized specifically for research purposes. All procedures complied with the ethical standards approved by the Faculty of Veterinary Medicine, Damanhour University (Approval No. DMU/VetMed-2023/050). This study was conducted and reported in accordance with the ARRIVE guidelines (https://arriveguidelines.org), ensuring ethical animal use, transparent reporting, and reproducibility in research involving animal tissues. The animals were sacrificed in Kom Hamada slaughterhouse in El Beheira Governorate, Egypt. In this study, camel’s brains were collected from ten clinically healthy mature camel’s (*Camelus dromedarius*) (4–6 years old) heads without sex differentiation. After slaughtering the camel, the entire head was separated from the neck, and saline was injected into the common carotid artery to flush the remaining blood from the head vasculature. The 4% paraformaldehyde in PBS was injected into the common carotid artery for at least 30 min, then the skull was opened to obtain the brain, and the cerebellum was carefully dissected from the brain. Every cerebellum was hemisected into two halves, and 4 sagittal cuts to each hemisphere, to permit more diffusion of formalin to the deepest regions, were performed and immersed in the same fixative at 4° C for 3 days, to ensure sufficient fixation of the central regions. The cerebella from seven camels were used for routine light microscopy and immunohistochemistry, and 3 animals were used for transmission electron microscopy (TEM) and semi-thin sectioning.

Three adult rats were also sacrificed by overdose of ketamine and perfused with saline. Their brains were collected and kept in Formalin 10% for 48 h. One hemisphere from each brain was dissected, processed for Paraffin embedding and cut by microtome at 5–7 μm thickness.

### Light microscopic study

The tissue blocks were prepared for paraffin cutting according to the standard methodology for histological and immunohistochemical studies. Sections were selected using a systematic random sampling approach: at least, 30 sagittal sections from the vermal region of folia VI-VIII were cut serially at 6–8 μm thickness using a Richert Leica RM 2125 Microtome, Germany, and mounted on glass slides. Every first section (*n* = 6) was stained with cresyl violet (to stain cell bodies) together with luxol fast blue (for myelinated fibers). Every second section (*n* = 6) was stained with Grimelius silver nitrate impregnation (for both cell bodies and processes).

### Semithin sections and transmission electron microscopy (TEM)

Karnovsky’s fixative was used to preserve small samples of cerebellar vermal tissues, which were kept at 4 °C for the entire night^66^. 10 mL of 25% paraformaldehyde, 10 mL of 50% glutaraldehyde, 50 mL of phosphate buffer, and 30 mL of distilled water were mixed to formulate Karnovsky’s fixative. The samples were postfixed for two hours at room temperature in 0.1 mol/L phosphate buffer and 1% osmic acid at pH 7.3. After being dehydrated and cleaned in increasing grades of ethanol and propylene oxide, respectively, the specimens were finally embedded in an Araldite-Epon mixture. A Reichert Ultracut (Leica, Germany) was used to cut semi-thin sections to a thickness of 1 μm. They were then stained with toluidine blue and examined with a LeitzDialux 20 microscope.

Using a Canon digital camera (Canon PowerShot A 95), images were captured. At the Assiut University Electron Microscopy Unit, ultrathin Sect.  (70 nm) were stained with uranyl acetate and lead citrate^67^ and analyzed using a JEOL 100CX II TEM (JEOL, Tokyo, Japan).

### Immunohistochemical staining

Every third section was mounted onto super frost slides for immunohistochemical staining using an UltraTek HRP Anti-Polyvalent (DAB) Staining System (ScyTek Laboratories, West Logan, UT, USA, AMF080) according to Mokhtar, et al.^68^. The sections were deparaffinized with xylene, rehydrated in graded ethanol, and washed with 0.1 M PBS (pH 7.4, twice × 5 min). To increase epitope exposure, was performed via boiling the slides for 10 min in a sodium citrate buffer (0.01 M, pH 6.0), and the sections were left at room temperature for 30 min to cool and washed with PBS. The endogenous peroxidase activity was quenched with 3% H_2_O_2_ in distilled water for 20 min at RT, followed by washing with PBS (2 *×* 5 min). The sections were blocked with the blocking solution of the kit for 5 min at RT. The sections were incubated overnight at 4 °C with the following primary antibodies: the diluted (1:100) primary antibodies against a rabbit polyclonal anti-S100 protein (1:100, Z0311, Dako, Glostrup, Denmark), a mouse monoclonal anti-Olig2 (1:100; Santa Cruz, Dallas, TX, USA, sc-515947), a the rabbit polyclonal anti-glial fibrillary acidic protein (GFAP) (1:400; PA5- 16291, Thermo Fisher Scientific, Waltham, MA, USA) and a rabbit polyclonal anti-Iba1 (1:500, 019-19741, Wako, Japan).

The used antibodies were validated before use, both by negative and positive control. to validate the secondary antibody, staining tissues without incubation with primary antibody and no reaction was seen. A positive control of the nervous tissue contains the specific antigen for each primary antibody, and we got a specific reaction (unpublished data). Rat sections were immunostained with GFAP, for comparing with camels’ cerebellum. Rat cerebellum section stained with GFAP in our own laboratory using identical immunohistochemical protocols to those applied to the camel tissue. It was generated as positive control, in a well-characterized rodent model, for the GFAP antibody and for comparative visualization.

Sections were rinsed (3 × 5 min each) with PBS and incubated for 15 min with the secondary Ultra Tek HRP Anti-polyvalent antibody (goat anti-mouse, rat, rabbit and guinea pig IgG) purchased from Scy Tek (TX, USA). Following that, the slides were washed three times for 3 min each with a PBS, and the tissues were incubated with the HRP for 15 min and then washed three times for 3 min each with a PBS. The visualization of the reaction was carried out with 3,3′diaminobenzidine (DAB) chromogen diluted with DAB substrate (provided within the same Scy Tek Detection kit) according to the manufacturer protocol for 10–15 min until the desired staining was achieved and counterstained with Harris hematoxylin. The sections were dehydrated in a graded series of ethanol, cleared with xylene mounted with mounting media, DPX. Immunohistochemical staining was evaluated by LeitzDialux Microscope, and photos were photographed by a Canon digital camera (Canon Powershot A95) in the Department of Anatomy and Histology, Faculty of Veterinary Medicine, Assiut University, Egypt.

### Morphometric analysis

The morphometric studies were performed on immunohistochemical images of the camel cerebellum using the Image-J program. Any quantified parameter was measured from 8 to 10 representative images taken from 10 sections per cerebellum of 5 animals. The results were presented as the mean of measurements + standard error and considered representative of these regions. The cellular density per Cross Sectional Area (CSA) was recorded by counting cells in the largest possible measured areas in different folia, which were of variable size. So, sometimes it was from the entire section and sometimes from most of it. Hence, after counting, the results were normalized to 1 square mm (1 mm^2^). The length of the Bergmann glia processes was measured using the *straight line* tool after calibration using the *set scale* tool from the *analyze* menu.

The rationale behind the sampling strategy to avoid bias were through (1) sampling from a fixed vermal region (folia VI–VIII) ensures anatomical consistency across animals; (2) systematic random field selection prevents operator bias in region-of-interest selection; and (3) measuring the largest possible areas within each folium and normalizing to 1 mm² minimizes the effect of variable section geometry.

**Fig. 13:**
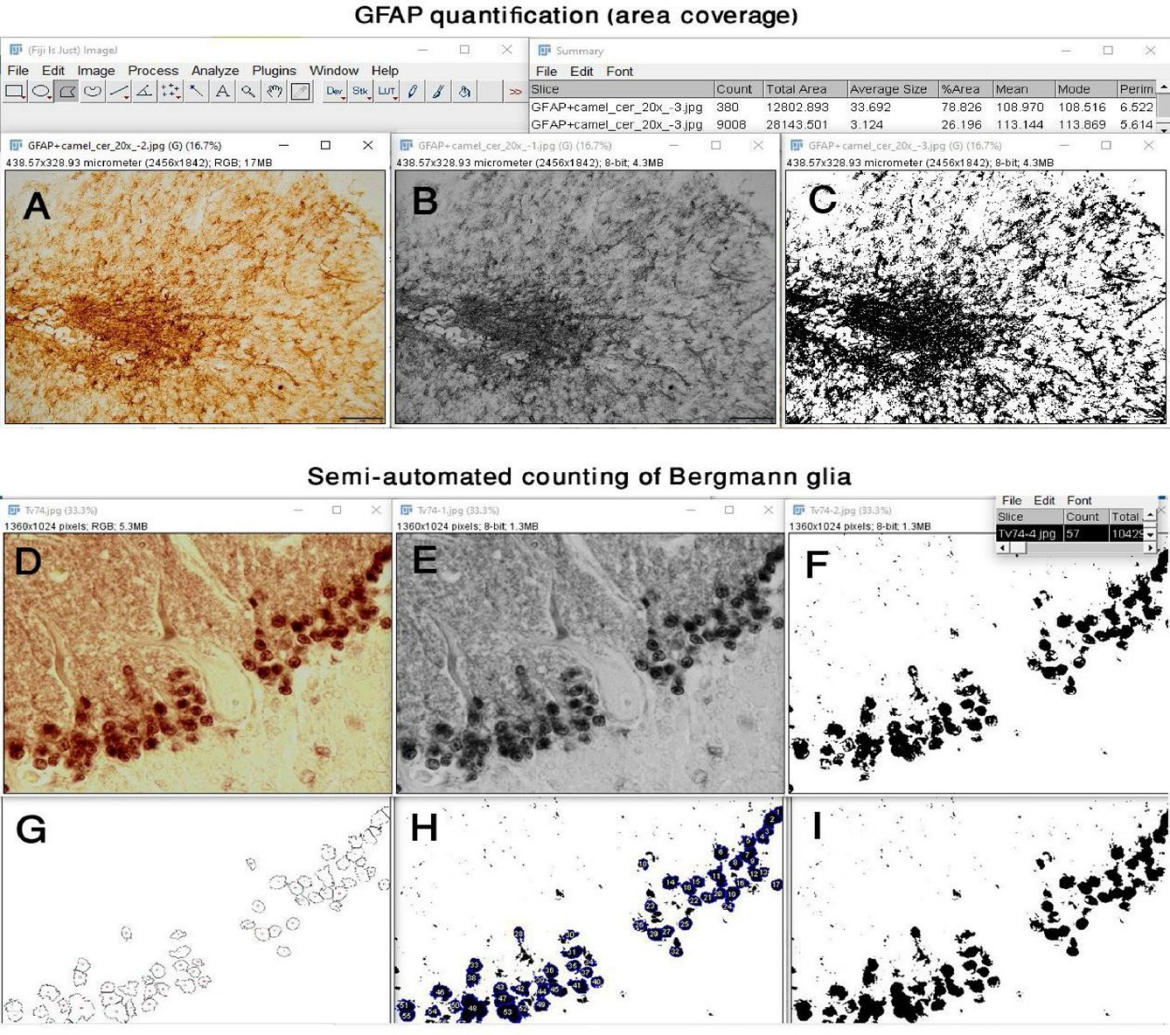
The steps of using ImageJ program for quantification of GFAP expression (area coverage, **A**-**C**) and Bergmann glia semiautomated counting (**D**-**I**). (**A**) the original image, (**B**) the image after converted to 8-bit, (**C**) the image after thresholding and adjust to the nearest view of the original image. (**D**) the original image, (**E**) the image after converted to 8-bit, (**F**) the image after thresholding and adjust to the nearest view of the original image. G) the overlay of the counted cells added by the ImageJ program, (**H**) the count number added by the ImageJ program for counted cells and I) the final shape of the counted cells.

The GFAP expression was quantified as the area coverage percentage, using the ImageJ program (Fig. 13 AC). The coverage areas were considered to be as large as possible in different folia which was not the same, so it was not a fixed areas to specify its dimensions. Moreover, the result of the coverage area calculated by the program as a percentage of immunopositive particles of the total analyzed area. Briefly, first, the picture was converted into an *8-bit* type from the *image* menu. Second, the *threshold* was chosen from *adjust* in the *image* menu. The threshold was done according to the default of the program, compared to the original-coloured image and adjusted to have the nearest view to the original ones. Finally, *analyze particles* was chosen from the *analyze* menu, and the reading *% area* was obtained from the *summary* window.

The Bergmann glia were counted using the semi-automated method of the ImageJ program (Fig. 13 DI). First, the picture was converted into an *8-bit* type from the *image* menu. Second, the *threshold* was chosen from the *adjust* in the *image* menu. The threshold was done according to the default of the program, compared to the original-coloured image and adjusted to have the nearest view to the original ones. Third, to separate the fused shadow of the adjacent cells, the pictures were converted into binary from the *binary* of the *process* menu, followed by choosing *watershed* from *binary*. Fourth, to restrict the counting to the cells only, but not other particles, the *area* of the smallest and largest Bergmann cells was measured to be used in the following step. Fifth, *analyze particles* was chosen from the *analyze* menu, and the smallest and largest areas were inserted in the size restriction option of the *analyze particles* small window, and the circularity was determined to be from 0.6 to 1, to avoid counting particles other than cells. Finally, the count was obtained from the *summary* window.

### Statistical analysis

The statistical tests applied to evaluate differences in cell densities across the various cerebellar layers in the current study were performed utilizing Graph Pad Prism 8 (GraphPad Software, Inc.) and presented as mean ± standard error of the mean (M ± SEM). The comparisons among different layers were performed using one-way analysis of variance (ANOVA) followed by Tukey’s post hoc test. When the p value was < 0.05 the comparisons were considered statistically significant.

## Data Availability

The datasets generated during and/or analysed during the current study are not publicly available but are available from the corresponding author on reasonable request.
